# Small-Scale Variations in Urban Air Pollution Levels Are Significantly Associated with Premature Births: A Case Study in São Paulo, Brazil

**DOI:** 10.3390/ijerph15102236

**Published:** 2018-10-12

**Authors:** Silvia Regina Dias Medici Saldiva, Ligia Vizeu Barrozo, Clea Rodrigues Leone, Marcelo Antunes Failla, Eliana de Aquino Bonilha, Regina Tomie Ivata Bernal, Regiani Carvalho de Oliveira, Paulo Hilário Nascimento Saldiva

**Affiliations:** 1 Centro de Pesquisa e Desenvolvimento para o SUS, Instituto de Saúde, Secretaria do Estado da Saúde de São Paulo, Rua Santo Antônio, 590-Bela Vista, São Paulo 01314-000, Brazil; 2 Departamento de Geografia da Faculdade de Ciências, Letras e Filosofia da Universidade de São Paulo, Cidade Universitária, Av. Prof. Luciano Gualberto—Butantã, São Paulo 05344-020, Brazil; lija@usp.br; 3 Instituto de Estudos Avançados da Universidade de São Paulo, Rua da Praça do Relógio, 109 andar Térreo. Cidade Universitária, São Paulo 05508-050, Brazil; pepino@usp.br; 4 Departamento de Pediatria da Faculdade de Medicina da Universidade de São Paulo, Av. Dr. Enéas de Carvalho Aguiar, 647-Cerqueira César, São Paulo 05403-000, Brazil; clearleone@uol.com.br; 5 Coordenação de Epidemiologia e Informação (CEInfo)—Secretaria Municipal da Saúde de São Paulo, R. General Jardim, 36-5º andar-Vila Buarque, São Paulo 01223-010, Brazil; marcelofailla@prefeitura.sp.gov.br (M.A.F.); ebonilha@prefeitura.sp.gov.br (E.d.A.B.); 6 Núcleo de Pesquisas Epidemiológicas em Nutrição e Saúde da Faculdade de Saúde Pública da Universidade de São Paulo, Av. Dr. Arnaldo, 715-Cerqueira César, São Paulo 01246-000, Brazil; rbernal@usp.br; 7 Laboratório de Poluição Ambiental do Departamento de Patologia da Faculdade de Medicina da Universidade de São Paulo, Av. Dr. Arnaldo, 455-Cerqueira César, São Paulo 01246-903, Brazil; regiani@usp.br

**Keywords:** premature birth, air pollution, air monitoring, spatial analysis

## Abstract

Premature birth is the result of a complex interaction among genetic, epigenetic, behavioral, socioeconomic, and environmental factors. We evaluated the possible associations between air pollution and the incidence of prematurity in spatial clusters of high and low prevalence in the municipality of São Paulo. It is a spatial case-control study. The residential addresses of mothers with live births that occurred in 2012 and 2013 were geo-coded. A spatial scan statistical test performed to identify possible low-prevalence and high-prevalence clusters of premature births. After identifying, the spatial clusters were drawn samples of cases and controls in each cluster. Mothers were interviewed face-to-face using questionnaires. Air pollution exposure was assessed by passive tubes (NO_2_ and O_3_) as well as by the determination of trace elements’ concentration in tree bark. Binary logistic regression models were applied to determine the significance of the risk of premature birth. Later prenatal care, urinary infection, and hypertension were individual risk factors for prematurity. Particles produced by traffic emissions (estimated by tree bark accumulation) and photochemical pollutants involved in the photochemical cycle (estimated by O_3_ and NO_2_ passive tubes) also exhibited significant and robust risks for premature births. The results indicate that air pollution is an independent risk factor for prematurity.

## 1. Introduction

Premature birth is the result of a complex interaction among genetic, epigenetic, behavioral, socioeconomic, and environmental factors [1,2]. Prematurity is associated with increased morbidity and mortality in the first year of life [3,4]. Published data indicate that prematurity enhances the risk of chronic diseases in adulthood [5] such as type 2 diabetes [6], respiratory disease [7], cardiovascular disease [8], and attention deficit disorders [9].

Exposure to environmental contaminants exhibits associations with premature births [10]. Because of its conspicuous nature, air pollution may be responsible for a considerable attributed fraction of global premature births [11]. Using global satellite-based estimates of exposure, Malley et al. [12] estimated that air pollution is responsible for 2.7 million premature births worldwide. Mechanisms responsible for the effects of air pollution exposure in premature birth are not fully clarified, but the induction of systemic inflammation [13] affects both fetal and placental homeostasis [14,15,16,17,18,19]. In addition, other determinants of prematurity may vary in space such as socioeconomic status (SES), demographics, housing characteristics, behavioral factors, and accessibility to health care. Thus, broader environmental properties may act to isolate or interact with air pollution to increase susceptibility of pregnant women and, consequently, affect birth outcomes [20,21,22].

The prevalence of prematurity in Brazil was 12.3% for the period from 2011 to 2012 [23] and were almost the same as in the Municipality of São Paulo for the period. Generally, premature births were more frequent in deprived areas where several social or environmental risk factors for prematurity co-existed [24]. We reasoned that further information about the role of air pollution in determining premature births could be obtained by evaluating possible associations between air pollution and the incidences of prematurity in spatial clusters of high and low prevalence in São Paulo. In this paper, we report the results of a case-control study conducted in three areas of São Paulo selected based on higher (2 spatial clusters) and lower prevalence (1 spatial cluster) of premature births using a combination of low-cost techniques designed to characterize the spatial variability of air pollution with high resolution. These results support the concept that particles and the ozone are significant risk factors for premature births in São Paulo.

## 2. Subjects and Methods

### 2.1. Definition of the Study

The present study has a case-control design.

### 2.2. Study Location

The municipality of São Paulo has a population of 12.2 million, which is distributed over 1.5 thousand square kilometers. Similar to other Latin American megacities, São Paulo has sharp social and economic contrasts that affect housing conditions and access to medical care. In the period evaluated in the present study (2012 and 2013), 348,337 live births occurred in São Paulo with an 11.9% prematurity rate that varied from 8.4% to 15.9% across São Paulo’s 96 administrative units.

### 2.3. Identification of Spatial Clusters of Prematurity

**Figure 1:**
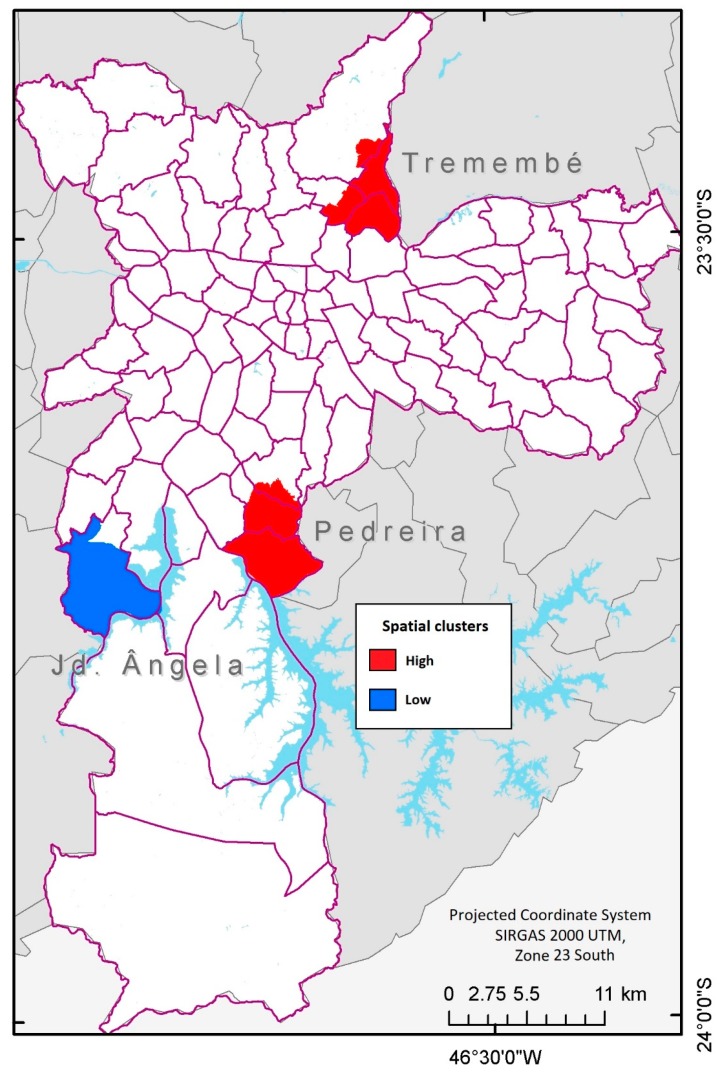
Spatial clusters of preterm deliveries in São Paulo, Brazil (2012–2013).

Data on live births and geo-coded residence addresses of the corresponding mothers were obtained from the Secretary of Health of the Municipality of São Paulo. The gestational period was computed for each live birth based on the mother’s day of last menstruation. Clusters of premature births were identified by applying a retrospective spatial scan statistical test using the software SaTScan^TM^ [25]. To minimize biases induced by grouping large administrative districts, cases were aggregated by using census units as geographic units because they presented significantly smaller and more homogeneous areas. Cluster identification was adjusted for the following covariates: mother’s age, type of pregnancy (singleton or multiple), and type of delivery (vaginal or caesarean). Cases were assumed to be poisson distributed with the constant risk over space under the null hypothesis in a bi caudal test. The spatial scan statistics arrange a circular window of variable size in the map surface and allows its center to move in such a way that, for a given position and size, the window includes a different set of near neighbors. If the window includes a neighbor centroid, the whole geographic unit is considered and included [25]. Cluster analysis results include spatial clusters with no geographic overlap of clusters allowed and a maximum allowable cluster size of 5% of the population. Significance was evaluated with Monte Carlo simulation with 999 replications where the null hypothesis of no clusters was rejected at an *α* level of 0.05. Such an approach, three clusters were identified with two of them with high prevalence (Tremembé and Pedreira) and one of them with low prevalence (Jardim Ângela) (Figure 1). High spatial clusters had an average radius of 3179 m from its center and the low cluster had a radius of 4848.7 m. Pedreira’s cluster included 2033 cases when 1836.38 were expected and Tremembé’s had 1274 cases when 1125.85 were expected. The low cluster in Jardim Ângela had 818 cases when 973.69 were expected.

### 2.4. Definition of Cases and Controls

**Figure 2:**
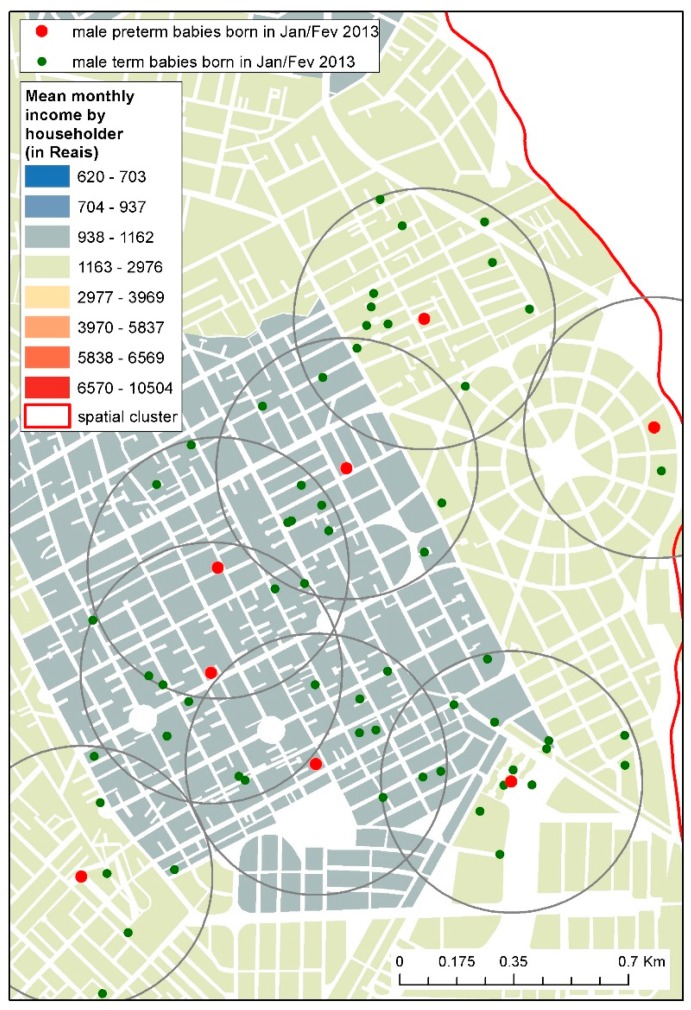
Example of spatial distribution of case-controls. The map depicts male term and preterm babies born in January and February 2013 and a 400 m-buffer from preterm babies overlaid on a map of mean monthly income by householder in a small portion of the municipality.

Cases were babies born with a gestational age of less than 37 weeks and controls were babies born at a gestational age equal to or more than 37 weeks. The sample was calculated based on the following: a paired study with a proportion of exposure between the cases of 40%, an Odds Ratio of 1.5 with a 10% significance level, and a power of the test of 80%. These assumptions indicated the necessity of 159 cases and 477 controls. For each case, two controls were randomly selected if they are the same sex and were born in the same or in the following month of cases’ date of birth and they lived no more than 400 m from the corresponding case (Figure 2). The following exclusion criteria were adopted: congenital malformations (Q00-Q99, ICD10, 1994), twins, and indigenous ethnicity.

### 2.5. Variables Related to Mothers

Since census units were considered the basis for geographic aggregation, secondary census information was included in the modeling such as the average number of residents per household, mean monthly income by householder (in Reais), percentage of households with bathrooms for the exclusive use of residents and sewage via general sewage systems, and percentage of dwellers according to ethnicity (black, mixed-race, and indigenous). Additionally, mothers were interviewed face-to-face by trained interviewers using questionnaires to gather personal information such as schooling, housing, employment, gestational history, pre-natal care, birth care, risk behavior for smoking, alcohol and drugs, and the presence of chronic disease. The interviews were based on five blocks comprising approximately 200 questions.

### 2.6. Estimates of Air Pollution Exposure

**Figure 3:**
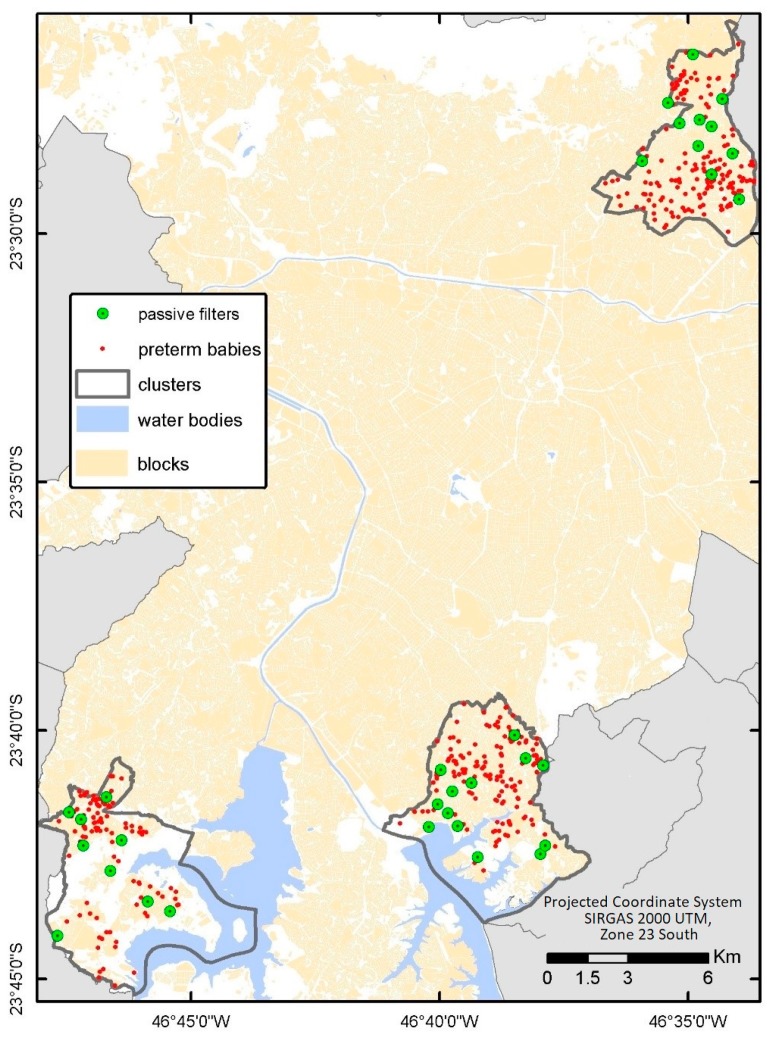
Spatial distribution of filters and interviewed preterm mothers in the studied clusters.

Two approaches were used to characterize exposure to air contaminants: passive tubes and the determination of trace element accumulation in tree bark. Passive tubes were used to measure nitrogen dioxide (NO_2_) and ozone (O_3_). The first pollutant is considered a proxy estimator of the gaseous component of fossil fuel burning while O_3_ reflects gaseous oxidants produced by photochemical processes. NO_2_ tubes were produced using filters impregnated with 2% triethanolamine, 0.05% o-metoxiphenol, and 0.025% sodium methabisulfite. The reaction with NO_2_ produced nitrite, which was measured by absorbance at 550 nm. For O_3_ measurements, we used cellulose filters impregnated with indigo carmine. After reacting with oxidants, the indigo was oxidized to isatin, which faded its blue coloration. The change in color was measured by optical reflectance. Filters in quadruplicate for each site were exposed for seven to 10 days, which provided the average concentration of the two pollutants in the time window of measurement. These two approaches were used by our group in previous field studies and exhibited good agreement with the same measures conducted by the State Sanitation Agency of São Paulo [26,27]. For each cluster, 10 filters were placed following a criterion of the proximity of cases/controls, which is shown in Figure 3. Measurements in each cluster were taken on four separate occasions that represented each season.

Trace elements in tree bark are indicative of particulate air pollution. This approach has previously been used in São Paulo and successfully provided small-scale discrimination of spatial variability of traffic-derived air pollution [28,29,30] as well as its source apportionment [21]. Particles emitted by pollution sources were trapped in the bark and represented a memory of particulate pollution that lasted for two years. Fragments of tree bark were collected and transformed into powder, the powder was compressed to form pastilles, and the pastilles were then analyzed by X-ray fluorescence spectroscopy. This was a multi-element procedure that allowed measurements of Al, Ba, Ca, Cl, Cu, Fe, K, Mg, Mn, Na, P, R, S, Sr, and Zn.

Based on the verification of existing trees in the areas of study, we collected samples from *Tipuana tipu*, *Caesalpinia pluviosa*, *Tibouchina granulosa*, and *Eucalyptus* sp. Similar to the passive tubes, 10 trees were sampled in each cluster.

Filters and trees were geo-coded and the attributed dose for each residence was extrapolated by using a quadratic spline based on the Euclidean distance between the residence and the spot of the air pollution measurement. In the statistical modeling, estimates of air pollution (passive tubes and tree bark bio-accumulation of trace elements) were considered categorical variables based on quartiles of the observed extrapolated dose for each child. For the passive tube measurements, the numerical values of the estimated concentration were used. For tree bark bio-accumulation, the elements were not considered individually. Rather, the coefficients of each factor attributed to every child were used.

### 2.7. Statistical Analysis

We used different statistical approaches. Geostatistical methods were described above. Factor analysis was used to group the elements measured in tree bark. Different sets of the considered elements were used to obtain the highest explained variance using the Varimax rotation. Descriptive statistics for the characteristics of the study population as well as a comparison between premature and term births among clusters were performed using univariate statistics. The significance of the risk of premature birth was determined by using binary logistic regression models that considered different sets of explanatory variables selected based on their significance detected in the univariate analysis. Although smoking did not reach statistical significance in the univariate models, it was included in the analysis because of the evidence in the literature on its role in favoring premature births. Calculations were performed using Excel v.10 (Microsoft, Redmond, WA, USA) and IBM SPSS v.13 (IBM Corporation, Armonk, NY, USA)for windows packages. Spatial analysis and mapping were performed using ESRI ArcGIS 10.1 (Esri, Redlands, CA, USA).

## 3. Results

The majority of the cases selected for the Tremembé cluster (110%) and Pedreira (96%) were studied and the lowest rate of success was achieved for Jardim Ângela (69%) mostly because of improper addresses (high relocation rates of the mothers) and other problems of access such as criminality. These three areas are, in fact, among the most deprived districts of São Paulo in terms of socio-economic indicators. The ratio between controls and cases in the three clusters was around 2:1.

Table 1 presents a summary of the characteristics of the mothers for each cluster and the statistical significance between premature and term births. Jardim Ângela had a high percentage of teenage mothers with premature babies (10%), which was higher than the other two clusters, and a high percentage of mothers without partners (35.5%). A high percentage of premature births for mothers with high levels of education were found in the Tremembé district.

Table 2 shows the characteristics of prenatal assistance in each cluster as well as the statistical significance between premature and term births. Mothers with preterm labor in the Pedreira district started prenatal care later (18.4%) and had a higher incidence of urinary infection (68.5%) and hypertension (63.2%) than mothers with term births had.

**Figure 4:**
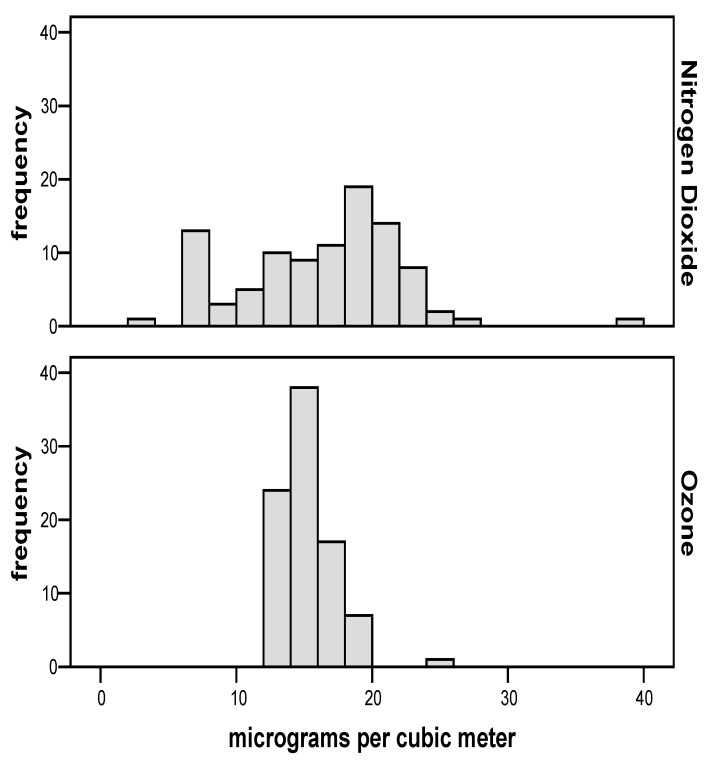
The estimated dose of NO_2_ and O_3_ for mothers enrolled in the study.

Figure 4 shows the estimated dose of NO_2_ and O_3_ for mothers enrolled in the study. Table 3 shows the descriptive statistics of trace element levels determined in tree bark.

Table 4 presents the results of the factor analysis that considered the elemental composition of tree bark. Four factors were identified that explained 82.8% of the variability. Factor 1 (Ba, Fe, Al, K, P, Cu, Rb, and Zn) and factor 2 (Mg, Mn, and S) were composed of trace elements associated with traffic emissions and soil suspension [31,32].

Table 5 shows the sensitivity analysis of the associations between estimates of air pollution and prematurity risk. Factor 1 was the only factor that exhibited robust dose-dependent associations with prematurity. The interaction term between high NO_2_ (fourth quartile) and low O_3_ (first quartile), the history of hypertension, urinary and syphilis infection during gestation, and the late onset of prenatal care exhibited significant positive risks for prematurity while low O_3_ (first quartile) was protective after controlling for the age of the mother and smoking. The associations of the estimators of air pollution exposure and risk of prematurity were sufficiently robust to remain stable across different model specifications.

## 4. Discussion

The present study detected significant associations between markers of exposure to ambient air pollution and the risk of premature births. Based on these results, variations in exposure in the microscale range determined by passive methods had an important influence on prematurity risk, which reinforces the concept that gestation represents a time window of extreme vulnerability to air pollution.

Although air pollution standards have been established by health authorities, it is quite possible that a safety threshold does not exist for airborne toxics. Because of their conspicuous presence in the urban environment, genetic, epigenetic profiles as well as comorbidities and social and economic determinants may increase the vulnerability of the exposed population to a point that even low concentrations may determine adverse health effects [33,34,35].

One of the main challenges in determining the adverse effects of air pollution on health, mainly in underprivileged populations, is the adequate characterization of small-scale variations of exposure [36,37]. In such context, we designed the present investigation by employing methods to capture small-scale variations of air contaminants and social and economic characteristics and aiming to determine whether air pollution has an independent role in determining higher risk for prematurity.

Epidemiologic studies conducted in different areas have reported significant associations between air pollution and prematurity [38]. Using variations in air pollution in the time domain, the period of gestation being more prone to the effects of air pollution exposure was explored by Rich et al. [39] and Giorgis-Allemand et al. [40]. The methods of pollution evaluation used in our investigation did not allow time resolution since they represented integrated measures across a period of time. Passive tubes averaged 10 days and four seasons while tree bark had a longer memory of trace element accumulation. The lack of time resolution was partly compensated by the high spatial resolution because the results allowed the determination of areas with different levels of contamination. Therefore, these determinations of exposure were rather qualitative but were useful to define areas with high and low pollution within the areas of study. Even considering the limitations of the exposure assessment, the results indicated that areas with higher levels of air pollution exhibited significantly higher risks of premature birth. The magnitude of the observed risks and the corresponding levels of significance were stable when different sets of controlling variables were included in the multivariate models. These findings suggest that the observed associations are sufficiently robust to model specifications that will provide additional support to our results.

It is important to notice that the measured concentrations of NO_2_ and O_3_ exhibited low concentrations of both contaminants, which was previously reported in other studies that focused on indoor air pollution conducted in São Paulo [41]. The same observation—low levels of pollution of NO_2_ and O_3_—were observed in a small pregnancy cohort conducted by our group [18]. It is important to mention that most of our population sample has their residencies in areas such as slums. In such a setting, the traffic is virtually negligible in the small pathways that cross the community. Thus, because of the distance from major roads, the concentrations of gaseous pollutants are probably attenuated by dispersion and dilution [28], but we cannot exclude the contribution of additional sources of indoor NO_2_. Butane, for instance, is the most used fuel for home cooking in our population and may have contributed, to some extent, to the observed NO_2_ levels detected by our passive tubes. However, our results indicate that traffic emissions are a significant source of air pollution in our study scenarios since the elements measured in tree bark are indicative of a significant presence of automotive emissions [31,32]. Since UV radiation is virtually absent in the indoor environment, we interpreted O_3_ levels measured by the passive tubes as the result of outdoor photochemical processes, which is likely derived from traffic sources. Lastly, filters measured the accumulated concentration from seven to 10 days, which is an event that could have dampened the variation of ambient concentrations of gaseous pollutants. Moreover, the initial idea—to measure the outdoor levels—was not possible since the filters were systematically vandalized. Thus, we had to install the filters indoors. This is a situation that most probably reduced even further the concentrations especially that of O_3_.

The results suggest that particles (estimated by tree bark accumulation) and photochemical pollutants involved in the photochemical cycle (estimated by O_3_ and NO_2_ passive tubes) play a role in the pathogenesis of premature birth (Table 5). The present study was not designed to investigate causal mechanisms. However, previous reports in the literature have described the potential mechanisms by which air pollution favors prematurity [42]. Placental insufficiency [14,15], trans placental transport of toxins [16], constriction of blood vessels in the umbilical cord [17], and alterations of placental flow [18] are examples of events associated with exposure to ambient levels of air pollution. Recent animal studies conducted by our group indicate that exposure to ambient levels of air pollution reduces the expression of angiotensin in placental tissue, which affects the invasion of trophoblast and, thus, reduces fetal-maternal interaction. The previously mentioned alterations, acting alone or in combination, indicate that exposure to air pollutants may create an unfavorable milieu for the fetuses up to the point of predisposition to prematurity [19].

This study also confirmed some characteristics classically associated with premature birth including urinary infection, arterial hypertension, and the late onset of prenatal care. In this context, our results indicate that a broad characterization of the urban environment including physical, social, cultural, and economic parameters is necessary to establish sound and efficient public policies that aim to reduce prematurity.

## 5. Conclusions

In conclusion, our results indicate that air pollution represents a significant risk for premature births. Intra-urban variations in exposure even at the scale of hundreds of meters may modify the risk. Additionally, this study suggests that low-cost techniques may be used to track the spatial variability of exposure and may be used in areas devoid of conventional pollution monitoring systems.

## Figures and Tables

**Table 1:** Distribution of the characteristics of the mothers in each cluster. The *p* values depicted in the first column represent the level of significance of the differences between preterm and term births.

Clusters	Mother’s Characteristics	Preterm				
		No	%	Yes	%	Total
**Tremembé**	<20 y	10	2.8	7	4.0	17
Age *p* = 0.2	20–34.9 y	256	71.7	113	64.2	369
	≥35 y	91	25.5	56	31.8	147
	Total	357	100	176	100	533
Ethnicity *p* = 0.3	White	175	49.0	76	43.2	251
	Black	48	13.4	21	11.9	69
	Asian	3	0.8	0	0	3
	Mixed	125	35.0	77	43.8	202
	Indigenous	6	1.7	2	1.1	8
	Total	357	100	176	100	533
Education *p* = 0.01	Elementary	92	25.8	30	17.1	122
	High school	203	56.9	99	56.6	302
	College	62	17.4	46	26.3	108
	Total	357	100	175	100	532
Civil status *p* = 0.2	Single	94	26.3	53	30.1	147
	Married	263	73.6	123	69.9	386
	Total	357	100	176	100	533
Residence time *p* = 0.28	<1 year	73	20.6	36	20.5	109
	1–5 years	130	36.6	76	43.2	206
	≥5 years	152	42.8	64	36.4	216
	Total	355	100	176	100	531
**Pedreira**	<20 y	21	5.9	7	4.5	28
Age *p* = 0.8	20–34.9 y	234	66.1	101	66.0	335
	≥35 y	99	27.9	45	29.4	144
	Total	354	100	153	100	507
Ethnicity *p* = 0.57	White	124	40.1	67	45.0	191
	Black	65	21.0	27	18.1	92
	Asian	2	0.6	0	0	2
	Mixed	118	38.2	55	36.9	173
	Indigenous	0	0	0	0	0
	Total	309	100	149	100	458
Education *p* = 0.87	Elementary	94	26.6	38	25.0	132
	High school	205	57.9	88	56.6	293
	College	55	15.5	26	17.1	81
	Total	354	100	152	100	506
Civil status *p* = 0.63	Single	129	36.4	50	32.7	179
	Married	225	63.6	103	67.3	328
	Total	354	100	153	100	507
Residence time *p* = 0.25	<1 year	32	9.2	21	13.8	53
	1–5 years	138	39.5	61	40.1	199
	≥5 years	179	51.3	70	46.1	249
	Total	349	100	152	100	501
**Jardim Ângela**	<20 y	8	3.0	11	10.1	19
Age *p* = 0.02	20–34.9 y	192	72.5	75	68.8	267
	≥35 y	65	24.5	23	21.1	88
	Total	265	100	110	100	374
Ethnicity *p* = 0.63	White	91	34.3	31	28.2	122
	Black	27	10.2	15	13.8	42
	Asian	1	0.4	1	0.9	2
	Mixed	145	54.7	61	56.0	207
	Indigenous	1	0.4	1	0.9	2
	Total	265	100	109	100	374
Education *p* = 0.43	Elementary	56	21.4	28	25.9	84
	High school	163	62.2	67	62.0	230
	College	43	16.4	13	12.0	56
	Total	262	100	108	100	370
Civil status *p* = 0.03	Single	75	28.3	39	35.8	114
	Married	190	71.7	70	64.2	260
	Total	265	100	109	100	374
Residence time *p* = 0.21	<1 year	34	12.8	21	19.3	55
	1–5 years	122	46.0	42	38.5	164
	≥5 years	109	41.1	46	42.2	155
	Total	265	100	109	100	374

**Table 2:** Distribution of prenatal characteristics in each cluster. The *p* values depicted in the first column represent the level of significance of the differences between preterm and term births.

Clusters	Prenatal Characteristics	Preterm				
		No	%	Yes	%	Total
**Tremembé**	1 trimester	310	87.6	151	87.7	461
Beginning prenatal care *p* = 0.7	2 trimester	39	11.0	17	9.9	56
	3 trimester	5	1.4	4	2.3	9
	Total	354	100	172	100	526
Formal work *p* = 0.38	Yes	198	55.8	95	54.0	293
	No	157	42.2	81	46.0	238
	Total	355	100	176	100	531
Public assistance *p* = 0.08	Yes	250	70.6	113	64.2	363
	No	104	29.4	63	35.8	167
	Total	354	100	176	100	530
Number of consultations *p* = 0.39	<7	74	21.1	39	22.5	113
	≥7	277	78.9	134	77.5	411
	Total	351	100	173	100	524
Urinary infection *p* = 0.91	Yes	206	58.0	103	58.5	309
	No	149	42.0	73	41.5	222
	Total	355	100	176	100	531
Hypertension *p* = 0.12	Yes	52	14.6	35	19.9	87
	No	303	85.4	141	80.1	444
	Total	355	100	176	100	531
Type of delivery *p* = 0.2	Vaginal	171	48.0	92	52.3	263
	C-section	185	52.0	84	47.7	269
	Total	356	100	176	100	532
**Pedreira**	1 trimester	304	91.8	124	81.6	428
Beginning prenatal care *p* = 0.01	2 trimester	26	7.9	23	15.1	49
	3 trimester	1	0.3	5	3.3	6
	Total	331	100	152	100	483
Formal work *p* = 0.53	Yes	177	50.7	77	50.7	254
	*No*	*172*	*49.3*	*75*	*49.3*	*247*
	Total	349	100	152	100	501
Public assistance *p* = 0.12	Yes	247	70.2	115	75.7	362
	No	105	29.8	37	24.3	142
	Total	352	100	152	100	504
Number of consultations *p* = 0.53	<7	89	25.5	38	25.3	127
	≥7	260	74.5	112	74.7	372
	Total	349	100	150	100	499
Urinary infection *p* = 0.001	Yes	29	8.3	63	41.4	92
	No	320	91.7	89	58.6	409
	Total	349	100	152	100	501
Hypertension *p* = 0.0001	Yes	21	6.0	36	23.7	57
	No	328	94.0	116	76.3	444
	Total	349	100	152	100	501
Type of delivery *p* = 0.32	Vaginal	197	55.6	81	52.9	278
	C-section	157	44.4	72	47.1	229
	Total	354	100	153	100	507
**Jardim Ângela**	1 trimester	241	90.9	93	86.1	334
Beginning prenatal care *p* = 0.18	2 trimester	22	8.3	15	13.9	37
	3 trimester	2	0.8	0	0	2
	Total	265	100	108	100	373
Formal work *p* = 0.41	Yes	112	42.3	44	40.4	156
	No	153	57.7	65	59.6	218
	Total	265	100	109	100	374
Public assistance *p* = 0.02	Yes	168	63.6	82	75.2	250
	No	96	36.4	27	24.8	123
	Total	264	100	109	100	373
Number of consultations *p* = 0.43	<7	53	20.0	20	18.5	73
	≥7	212	80.0	88	81.5	300
	Total	265	100	108	100	373
Urinary infection *p* = 0.44	Yes	155	58.5	59	54.1	214
	No	110	41.5	50	45.9	160
	Total	265	100	109	100	374
Hypertension *p* = 0.97	Yes	46	17.4	19	17.4	65
	No	219	82.6	90	82.6	309
	Total	265	100	109	100	374
Type of delivery *p* = 0.34	Vaginal	126	47.5	55	50.5	181
	C-section	139	52.5	54	49.5	193
	Total	265	100	109	100	374

**Table 3:** Descriptive statistics (minimum, maximum, mean, and std. deviation) of trace element levels determined in tree bark (ppm).

Elements	Minimum	Maximum	Mean	Std. Deviation
Al	66.71	3873.10	571.52	500.98
Ba	59.55	1736.03	325.65	219.68
Ca	9985.05	39,883.30	25,167.06	4984.49
Cl	29.59	772.51	144.19	64.43
Cu	3.97	7.44	4.61	0.39
Fe	115.79	3630.08	644.44	465.86
K	540.46	8167.26	1998.30	904.04
Mg	496.37	4442.16	1405.33	462.27
Mn	18.48	1487.87	113.26	142.33
Na	8.05	22.20	16.89	1.95
P	367.18	1682.65	738.05	171.59
Rb	7.04	24.76	12.28	1.83
S	805.46	3699.55	1842.72	433.43
Sr	27.74	159.26	78.75	17.63
Zn	10.71	126.39	55.20	23.33

**Table 4:** Rotated matrix solution of elemental composition based on tree bark bioaccumulation studies.

COMPONENT MATRIX				
ELEMENTS	Factor 1	Factor 2	Factor 3	Factor 4
CU	0.686	0.140	0.485	−0.167
CA	−0.362	−0.621	0.339	0.485
K	0.773	0.039	0.542	−0.045
CL	0.426	0.240	0.290	−0.462
S	0.298	0.582	0.459	0.442
P	0.760	0.071	0.482	0.080
AL	0.786	−0.074	−0.552	0.184
MG	−0.224	0.794	−0.014	−0.324
NA	−0.568	−0.674	0.336	−0.131
BA	0.844	0.083	−0.437	0.206
SR	−0.275	0.412	0.425	0.711
RB	0.549	−0.298	0.468	−0.278
ZN	0.519	−0.722	0.017	0.208
MN	−0.333	0.731	−0.057	0.157
FE	0.808	−0.003	−0.536	0.192

Extraction Method: Principal Component Analysis.

**Table 5:** Multivariate logistic model with preterm and variables related to air pollution, the characteristics of mothers, and the onset of prenatal assistance.

Models	Variables	Exp (B)	*p*	Lower CI 95%	Upper CI 95%
Model 1—Pollutants	Low NO_2_	1.03	0.98	0.76	1.33
	Low O_3_	0.50	0.001	0.36	0.69
	Factor 1 (level 2)	0.91	0.60	0.65	1.28
	Factor 1 (level 3)	1.51	0.02	1.08	2.12
	Factor 1 (level 4)	1.73	0.004	1.19	2.50
Model 2—Pollutants and mothers’ characteristics	Low NO_2_	0.99	0.96	0.75	1.32
	Low O_3_	0.51	0.001	0.37	0.70
	Factor 1 (level 2)	0.89	0.53	0.64	1.26
	Factor 1 (level 3)	1.52	0.02	1.08	2.13
	Factor 1 (level 4)	1.72	0.004	1.18	2.49
	Mother’s age (<19 y)	1.50	0.14	0.87	2.58
	Mother’s age (>34 y)	1.10	0.47	0.85	1.43
	High school level	1.20	0.21	0.90	1.60
	University level	1.32	0.14	0.91	1.90
Model 3—Pollutants, mothers’ characteristics, smoking, use of drugs, and prenatal disease	Low NO_2_	0.86	0.33	0.63	1.16
	Low O_3_	0.46	0.001	0.33	0.65
	Factor 1 (level 2)	0.87	0.43	0.60	1.24
	Factor 1 (level 3)	1.60	0.01	1.12	2.29
	Factor 1 (level 4)	1.65	0.01	1.11	2.45
	Mother’s age (<19 y)	1.41	0.45	0.79	2.51
	Mother’s age (>34 y)	1.11	0.62	0.84	1.47
	High school level	1.25	0.16	0.92	1.70
	University level	1.52	0.05	0.99	2.31
	Public assistance	1.34	0.05	1.00	1.80
	Use of drugs	1.13	0.80	0.43	2.98
	Smoking	0.79	0.28	0.51	1.22
	Alcohol consumption	0.91	0.70	0.55	1.50
	Urinary infection	1.69	0.001	1.31	2.19
	Hypertension	1.71	0.001	1.23	2.38
	Syphilis	5.02	0.001	1.93	13.05
	2nd trimester onset of prenatal care	1.74	0.001	1.26	2.39
	3rd trimester onset of prenatal care	1.18	0.72	0.47	2.98

Low O_3_ is the first quartile and comprises values ≤ 14.2 μg/m^3^ and high NO_2_ ≥ 16.4 μg/m^3^.

## References

[1] Goldenberg R.L., Culhane J.F., Iams J.D., Romero R. (2008). Epidemiology and causes of preterm birth. Lancet.

[2] Gravett M.G., Rubens C.E., Nunes T.M. (2010). Global report on preterm birth and stillbirth (2 of 7): Discovery science. BMC Pregnancy Childbirth.

[3] Beck S., Wojdyla D., Say L., Betran A.P., Merialdi M., Requejo J.H., Rubens C., Menon R., Van Look P.F. (2010). The worldwide incidence of preterm birth: A systematic review of maternal mortality and morbidity. Bull. World Health Organ..

[4] Henderson J.J., McWilliam O.A., Newnham J.P., Pennell C.E. (2012). Preterm birth aetiology 2004–2008. Maternal factors associated with three phenotypes: Spontaneous preterm labour, preterm pre-labour rupture of membranes and medically indicated preterm birth. J. Matern. Fetal Neonatal Med..

[5] Visentin S., Grumolato F., Nardelli G.B., Di Camillo B., Grisan E., Cosmi E. (2014). Early origins of adult disease: Low birth weight and vascular remodeling. Atherosclerosis.

[6] Li S., Zhang M., Tian H., Liu Z., Yin X., Xi B. (2014). Preterm birth and risk of type 1 and type 2 diabetes: Systematic review and meta-analysis. Obes. Rev..

[7] O’Reilly M., Sozo F., Harding R. (2013). Impact of preterm birth and bronchopulmonary dysplasia on the developing lung: Long-term consequences for respiratory health. Clin. Exp. Pharmacol. Physiol..

[8] Bayman E., Drake A.J., Piyasena C. (2014). Prematurity and programming of cardiovascular disease risk: A future challenge for public health. Arch. Dis. Child. Fetal Neonatal Ed..

[9] Huang J., Zhu T., Qu Y., Mu D. (2016). Prenatal, perinatal and neonatal risk factors for intellectual disability: A systemic review and meta analysis. PLoS ONE.

[10] Burris H.H., Baccarelli A.A., Wright R.O., Wright R.J. (2016). Epigenetics: Linking social and environmental exposures to preterm birth. Pediatr. Res..

[11] Stieb D.M., Chen L., Eshoul M., Judek S. (2012). Ambient air pollution, birth weight and preterm birth: A systematic review and meta-analysis. Environ. Res..

[12] Malley C.S., Kuylenstierna J.C., Vallack H.W., Henze D.K., Blencowe H., Ashmore M.R. (2017). Preterm birth associated with maternal fine particulate matter exposure: A global, regional and national assessment. Environ. Int..

[13] Shah P.S., Balkhair T., Knowledge Synthesis Group on Determinants of Preterm/LBW births (2011). Air pollution and birth outcomes: A systematic review. Environ. Int..

[14] Veras M.M., Damaceno-Rodrigues N.R., Caldini E.G., Ribeiro A.A.M., Mayhew T.M., Saldiva P.H., Dolhnikoff M. (2008). Particulate urban air pollution affects the functional morphology of mouse placenta. Biol. Reprod..

[15] Kampa M., Castanas E. (2008). Human health effects of air pollution. Environ. Pollut..

[16] Van den Hooven E.H., Pierik F.H., de Kluizenaar Y., Hofman A., van Ratingen S.W., Zandveld P.Y., Russcher H., Lindemans J., Miedema H.M., Steeqers E.A. (2012). Air pollution exposure and markers of placental growth and function: The generation R study. Environ. Health Perspect..

[17] Novack L., Yitshak-Sade M., Landau D., Kloog I., Sarov B., Karakis I. (2016). Association between ambient air pollution and proliferation of umbilical cord blood cells. Environ. Res..

[18] Carvalho M.A., Bernardes L.S., Hettfleisch K., Pastro L.D., Vieira S.E., Saldiva S.R., Saldiva P.H., Francisco R.P. (2016). Associations of maternal personal exposure to air pollution on fetal weight and fetoplacental Doppler: A prospective cohort study. Reprod. Toxicol..

[19] Vadillo-Ortega F., Osornio-Vargas A., Buxton M.A., Sánchez B.N., Rojas-Bracho L., Viveros-Alcaráz M., Castillo-Castrejón M., Beltrán-Montoya J., Brown D.G., O’Neill M.S. (2014). Air pollution, inflammation and preterm birth: A potential mechanistic link. Med Hypotheses.

[20] Hajat A., Hsia C., O’Neill M.S. (2015). Socioeconomic disparities and air pollution exposure: A global review. Curr. Environ. Health Rpt..

[21] Tu J., Tu W., Tedders S.H. (2016). Spatial variations in the associations of term birth weight with ambient air pollution in Georgia, USA. Environ. Int..

[22] Coker E., Liverani S., Su J.G., Molitor J. (2018). Multi-pollutant modeling through examination of susceptible subpopulations using profile regression. Curr. Environ. Health Rep..

[23] Passini R., Cecatti J.G., Lajos G.J., Tedesco R.P., Nomura M.L., Dias T.Z., Haddad S.M., Rehder P.M., Pacagnella R.C., Costa M.L. (2014). Brazilian Multicentre Study on Preterm Birth (EMIP): Prevalence and factors associated with spontaneous preterm birth. PLoS ONE.

[24] Behrman R.E., Butler A.S., Institute of Medicine (2004). Preterm Birth: Causes, Consequences, and Prevention.

[25] SaTScanTM, v9.3; Boston, MA, USA, 2014. http://www.satscan.org/.

[26] Novaes P., Saldiva P.H.N., Kara-José N., Macchione M., Matsuda M., Racca L., Berra A. (2007). Ambient levels of air pollution induce goblet-cell hyperplasia in human conjunctival epithelium. Environ. Health Perspect..

[27] Saldiva de André C.D., de André P.A., Rocha F.M., Saldiva P.H.N., Oliveira R.C., Singer J.M. (2014). Reliability of reflectance measures in passive filters. Atmos. Environ..

[28] Carneiro M.F.H., Ribeiro F.Q., Fernandes-Filho F.N., Lobo D.J.A., Barbosa F., Rhoden C.R., Mauad T., Saldiva P.H.N., Carvalho-Oliveira R. (2011). Pollen abortion rates, nitrogen dioxide by passive diffusive tubes and bioaccumulation in tree barks are effective in the characterization of air pollution. Environ. Exp. Bot..

[29] Amato-Lourenco L.F., Lobo D.J.A., Guimarães E.T., Moreira T.C.L., Carvalho-Oliveira R., Saiki M., Saldiva P.H.N., Mauad T. (2017). Biomonitoring of genotoxic effects and elemental accumulation derived from air pollution in community urban gardens. Sci. Total Environ..

[30] Carvalho-Oliveira R., Amato-Lourenço L.F., Moreira T.C., Silva D.R.R., Vieira B.D., Mauad T., Saiki M., Saldiva P.H.N. (2017). Effectiveness of traffic-related elements in tree bark and pollen abortion rates for assessing air pollution exposure on respiratory mortality rates. Environ. Int..

[31] Moreira T.C.L., de Oliveira R.C., Amato-Lourenço L.F., Kang C.M., Saldiva P.H.N., Saiki M. (2016). Intra-urban biomonitoring: Source apportionment using tree barks to identify air pollution sources. Environ. Int..

[32] Moreira T.C.L., Amato-Lourenço L.F., da Silva G.T., Saldiva de André C.D., de André P.A., Barrozo L.V., Singer J.M., Saldiva P.H.N., Saiki M., Locosselli G.M. (2018). The use of tree barks to monitor traffic related air pollution: A case study in São Paulo–Brazil. Front. Environ. Sci..

[33] Schwartz J., Laden F., Zanobetti A. (2002). The concentration-response relation between pm (2.5) and daily deaths. Environ. Health Perspect..

[34] Makri A., Stilianakis N.I. (2008). Vulnerability to air pollution health effects. Int. J. Hyg. Environ. Health.

[35] Chen G., Guo Y., Abramson M.J., Williams G., Li S. (2018). Exposure to low concentrations of air pollutants and adverse birth outcomes in Brisbane, Australia, 2003–2013. Sci. Total Environ..

[36] Brindley P., Maheswaran R., Pearson T., Wise S., Haining R.P., Maheswaran R., Craglia M. (2004). Using modeled air pollution data for health surveillance. GIS in Public Health Practice.

[37] HEADLAMP, World Health Organization, Office of Global and Integrated Environmental Health, United Nations Environment Programme & United States Environmental Protection Agency. Linkage Methods for Environment and Health Analysis: General Guidelines: A Report of the Health and Environment Analysis for Decision-making (HEADLAMP) Project. http://www.who.int/iris/handle/10665/62988.

[38] Fleischer N.L., Merialdi M., van Donkelaar A., Vadillo-Ortega F., Martin R.V., Betran A.P., Souza J.P. (2014). Outdoor air pollution, preterm birth, and low birth weight: Analysis of the world health organization global survey on maternal and perinatal health. Environ. Health Perspect..

[39] Rich D.Q., Liu K., Zhang J., Thurston S.W., Stevens T.P., Pan Y., Kane C., Weinberger B., Ohman-Strickland P., Woodruff T.J., Duan X. (2015). Differences in birth weight associated with the 2008 Beijing Olympics air pollution reduction: Results from a natural experiment. Environ. Health Perspect..

[40] Giorgis-Allemand L., Pedersen M., Bernard C., Aguilera I., Beelen R.M., Chatzi L., Cirach M., Danileviciute A., Dedele A., van Eijsden M. (2017). The influence of meteorological factors and atmospheric pollutants on the risk of preterm birth. Am. J. Epidemiol..

[41] Vieira S.E., Stein R.T., Ferraro A.A., Pastro L.D., Pedro S.S., Lemos M., da Silva E.R., Sly P.D., Saldiva P.H. (2012). Urban air pollutants are significant risk factors for asthma and pneumonia in children: The influence of location on the measurement of pollutants. Arch. Bronconeumol..

[42] Soto S.F., Melo J.O., Marchesi G.D., Lopes K.L., Veras M.M., Oliveira I.B., Souza R.M., de Castro I., Furukawa L.N.S., Saldiva P.H.N. (2017). Exposure to fine particulate matter in the air alters placental structure and the renin-angiotensin system. PLoS ONE.

